# Upregulation of *CDC7* Associated with Cervical Cancer Incidence and Development

**DOI:** 10.1155/2021/6663367

**Published:** 2021-03-03

**Authors:** Qingwei Wang, Weiping Zheng

**Affiliations:** Department of Gynecology and Obstetrics, Shaoxing People's Hospital, Shaoxing Hospital, Zhejiang University School of Medicine, Shaoxing, China

## Abstract

**Background:**

Cervical cancer is a common malignant tumor of women. Using integrated bioinformatics, this study identified key disease-causing genes in cervical cancer that may provide effective biomarkers or therapeutic targets for early diagnosis and treatment.

**Results:**

We used high-throughput sequencing data from the Gene Expression Omnibus (GEO) to identify new cervical cancer biomarkers. The GSE63678 dataset was downloaded. The data was analyzed via bioinformatics methods, and 61 differentially expressed genes were obtained. These differential genes were analyzed by the Gene Ontology (GO) and Kyoto Encyclopedia of Genes and Genomes (KEGG) pathway enrichments analyses. GO analysis demonstrated that the basic biological functions of differential genes were mostly regulating cell division, mitotic nuclear division, and immune response. Analysis of the KEGG pathway showed the primary involved in the cell cycle, p53 signaling pathway, and cytokine-cytokine receptor interactions. Using TCGA database to query differential expression of differential genes in cervical cancer, the *CDC7* gene was found to be highly expressed. *In silico* analysis of protein interactions using the STRING database revealed that *CDC7* interacts with many proteins. These findings were then validated *in vitro* with immunohistochemistry and qRt-PCR to confirm that *CDC7* is highly expressed in cervical cancer tissues. Cell function tests demonstrated that inhibition of *CDC7* expression could inhibit the proliferation and migration of cervical cancer HeLa and SiHa cells and promote apoptosis.

**Conclusion:**

With comprehensive bioinformatics combined with clinical and cellular function analysis, *CDC7* is important to the development of cervical cancer. Targeting of this biomarker may improve the early diagnosis and treatment of cervical cancer.

## 1. Introduction

Cervical cancer, ranked second among female malignancies (the first is breast cancer), has more than 1.5 million newly diagnosed cases each year and a death toll exceeding 300,000 [1, 2]; it is therefore a serious health problem worldwide [1]. Early detection of cervical cancer leaves a possibility for cure, but advanced, and persistent or recurrent cervical cancer is usually difficult to treat and is basically incurable. Although there are some effective preventive measures for cervical cancer, it is still one of the leading causes of cancer-related death in women under 60 years of age [1, 3, 4]. Persistent human papillomavirus (HPV) infection is one condition for the development of cervical cancer, but the occurrence of cervical cancer is caused by a combination of factors, such as social factors, genetic susceptibility factors, physical factors, and biological factors. Currently, surgical resection is still the preferred treatment, but it may be possible to better treat cervical cancer with other treatments. Therefore, research into the molecular mechanism of occurrence and development of cervical cancer, reliable as well as effective molecular markers, and more valid methods to obstruct and control the metastasis and proliferation of tumor cells as well as to promote the apoptosis of tumor cells is vital.

Here, we searched the differential genes in cervical cancer using the GEO database and selected the differentially expressed gene *CDC7*. Cell division cycle 7 (*CDC7*) is a serine/threonine kinase that plays a key role in the initiation of DNA replication and in G1/S phase transitions (cell cycle checkpoints) [5]. Moreover, *CDC7* plays a different role in different types of DNA replication. For example, *CDC7* in some cell types promotes the initial phase of the cell cycle; on the other hand, it is the ultimate inactive target of cell cycle checkpoints in other cell types [6, 7]. Overexpression of *CDC7* appears to be associated with several cancers caused by inhibition of p53 expression [6].

The expression of differential *CDC7* in cervical cancer and normal cervical tissues was compared by immunohistochemistry and extraction of RNA and quantitative reverse transcription-polymerase chain reaction (qRT-PCR). The role of *CDC7* in cervical cancer HeLa and SiHa cell lines was verified by cell functional experiments. Experimental results can confirm that *CDC7*, as an oncogene, plays a significative role in the progression as well as development of cervical cancer.

## 2. Materials and Methods

### 2.1. Collection of Data from GEO and Identification of Differential Genes

The GEO database is a common functional genome dataset, which allows the user to download established gene expression profiles. We searched the GEO datasets (http://www.ncbi.nlm.nih. gov/GEO/) for keywords, including “cervical cancer”; “Normal”; and “tissue” or “tumor” or “cancer” or “carcinoma” or “carcinoma” or “neoplasm.” Data of the disease group and control group were analyzed retrospectively. Then, download the CEL file for follow-up analysis, which contains 5 cases of normal cervical tissue as well as 5 cases of cervical cancer tissue. The platform for GPL571 is [HG-U133A_2] Affymetrix Human Genome U133A 2.0 Array. All data were statistically and graphically processed using the R software (https://www.r-project.org/), and converted and unqualified data was removed, using the affy package (http://www. http://bioconductor.org/packages/release/bioc/html/affy.html) load CEL file expression data. Additionally, the limma package (http://www.bioconductor.org/packages/release/bioc/html/limma.html) was employed to process data and standardize it. These genes were finally sequenced according to the value of log fold change where RANK analysis (adjusted *P* value <0.05) using the Bonferroni correction method was utilized. The null hypothesis of the rank method is that each gene is randomly classified in each experiment; the smaller the *P* value, the higher the ranking.

### 2.2. Functional and Pathway Analysis

The functions and pathways of differentially encoded proteins were analyzed online and annotated [8]. GO annotation used the DAVID tool to perform online (https://david.ncifcrf.gov/) on screened differentially expressed genes (DEGs), using the KOBAS online analysis database (available online: http://kobas.cbi.pku.edu.cn/) of DEGs at KEGG pathway analysis. We analyzed the significantly upregulated and downregulated DEGs measured by integrated microarray data, A *P* value of <0.05 was considered statistically significant. In this data study, we analyzed significant up- and downregulation of DEGs detected from integrated microarray data of cervical cancer. A *P* value of <0.05 was considered statistically significant.

### 2.3. Query of Differential Expression Using TCGA Database

The Cancer Genome Atlas (TCGA) database (gepia.cancer-pku.cu) was used to query the above analysis and the differential expression of DEGs in cervical cancer.

### 2.4. Protein Interaction Network

The STRING database (http://string-db.org/) is a platform that can be searched for experimental data, text mining, and predictive bioinformatics between known proteins and predicted proteins to identify interactions [9]. In the resulting analysis, every node is a protein, molecule, or gene, and these connections represent the interactions between those biomolecules. It can be used to confirm the interactions and pathways between proteins encoded by DEGs in cervical cancer.

### 2.5. Patients and Samples

The 30 archived wax blocks (originally collected from January 1, 2019, to January 1, 2020) were analyzed with immunohistochemistry. The samples contained 15 normal cervix and 15 cervical cancer tissues. Further, 15 cases of fresh cervical cancer tissue and 15 cases of normal cervical tissue (originally collected from January 1, 2019, to January 1, 2020). No relevant treatment was performed before sampling.

### 2.6. Immunohistochemical Staining

Tissue samples were routinely dehydrated and embedded. They were then cut into 5 *μ*m serial sections and dewaxed with xylene for 5 min which was repeated three times. After ethanol, gradient hydration and sections were incubated with 3% hydrogen peroxide solution for 10 min at room temperature. Then, they were rinsed with phosphate buffer solution (PBS) for 5 min repeated three times. A solution of 5% bovine serum albumin (BSA) was added dropwise to the cells, and they were incubated at 37°C for 30 min. The primary antibody (Bioss, BJ, China) was added dropwise at a concentration of 1 : 100, and they were incubated at 4°C overnight and rinsed with PBS for 5 min, repeated three times, and the secondary antibody was added for incubation for 30 min at room temperature. The solution was colored with DAB solution to observe the color reaction under the microscope. After washing in running water, the cells were hematoxylin counterstained. The PBS was used instead of the primary antibody as a negative control, and the cervical cancer tissue with known *CDC7* positive expression was used as a positive control. The expression level of the *CDC7* protein is expressed by an integrated optical density (IOD) value.

### 2.7. qRT-PCR

Fresh cervical tissue was collected from patients with and without cervical cancer who required total hysterectomy. Total RNA was extracted with Trizol reagent according to the manufacturer's instructions, with appropriate dilution to an approximately equal concentration.

According to the preparation instructions of cDNA (FastQuant cDNA first strand synthesis kit), the prepared cDNA is based on 2 *μ*l total RNA. According to the SYBR Green protocol, a 20 *μ*l total reaction mixture and RNA7500 real-time PCR System (Applied Biosystems, CA, USA) were prepared to detect differences in the expression of *CDC7* at the RNA level (si*CDC7* (sense 5′-ATGCCTTGGTAGACTTTGGTTTG-3′; antisense 5′-GTTTCCTGTGATTATGTGGGATTTG-3′) and its negative controls).

### 2.8. Cell Culture and Transfection

The cervical cancer cell lines, HeLa and SiHa, were purchased from the cell bank at the Chinese Academy of Sciences (Shanghai). They were cultured in 10% fetal bovine serum (FBS, Gibco, MD, USA) 1640 medium (Gibco, MD, USA) in 5% CO_2_ and 37°C. Lipofectamine 2000 (Invitrogen, CA, Carlsbad) was used for transfection according to established protocols.

### 2.9. Cell Counting Kit-8 (CCK-8)

Human cervical cancer HeLa and SiHa cells grew to logarithmic growth phase. Then, cells were collected, and the solution was adjusted in concentration to 3 × 10^4^ cells/ml. Then, 100 *μ*l of the cell suspension was inoculated into each well in a 96-well plate, which was then cultured in a 5% CO_2_ at 37°C incubator for 0, 24, 48, and 72 hours. Finally, 10 *μ*l of CCK-8 (BestBio, SH, China) was added to each well of the 96-well plates. After the reagent was cultured for 3 hours, the optical density (OD) values were measured at a wavelength range of 450 nm. This experiment was repeated three times to detect the proliferation ability of cells after transfection.

### 2.10. Transwell Chamber Experiment

BD Matrigel Matrigel was diluted according to the instructions, gently spread on the Transwell (Corning Costar, USA) upper chamber, and placed in a 24-well plate. The lower chamber contained 500 *μ*l of medium containing 20% FBS, and the upper chamber was 200 *μ*l of a cell suspension having a concentration of about 2 × 10^5^/ml, and three replicate wells were set for each group. After culturing for 24 h, they were removed from the Transwell chamber, and the excess cells were carefully wiped off on the surface of the upper chamber membrane and rinsed twice with PBS; then, fixation was continued at room temperature for 30 min with paraformaldehyde. Crystal violet dye was employed for 20 min incubation and then rinsed off several times with distilled water. The cells in the microporous membrane were counted with an inverted microscope. Each sample was selected from five relatively average fields of view, the number of cells in each field of view was counted, and then, the average value was calculated.

### 2.11. Flow Cytometry for Apoptosis

HeLa cells were plated into 6-well plates for 24 h, and siRNA and negative controls were added. After 48 h, each group of cells was collected. Then, 5 *μ*l of Annexin V-FITC (BestBio, SH, China) was added to 500 *μ*l of cell suspension and incubated at 4°C for 15 min in the dark, and then 5 *μ*l of propidium iodide (PI, BestBio, SH, China) was added. The mixture was incubated at 4°C for 5 min again in the dark. Upstream cytometry was used to detect the apoptosis rate of each group.

### 2.12. Statistical Analysis

All data analysis and mapping used GraphPad Prism 8. SPSS 25.0 was used to obtain *P* values for statistical analysis. The results were expressed using x ± s. *P* < 0.05 indicated statistical significance.

## 3. Result

### 3.1. Bioinformatic Analysis of Differential Genes

#### 3.1.1. Screening of Differential Genes

**Figure 1:**
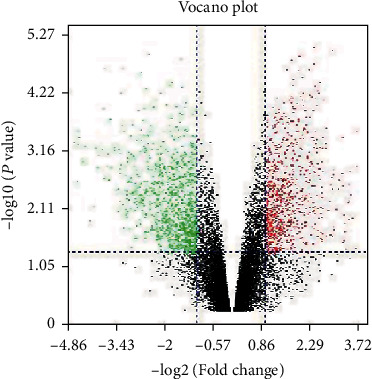
Differential expression of data. The red points on behalf of upregulated genes sized on the basis of ∣fold change | >2.0 and corrected *P* value of <0.05. The green points on behalf of downregulation of the expression of genes sized on the basis of ∣fold change | >2.0 and corrected *P* value of <0.05. The black points represent with no observably difference genes.

The GSE63678 dataset, containing 10 samples, including 5 cervical cancer samples and 5 normal cervical samples, was downloaded. The cervical cancer expression chip dataset was screened by the limma package in the R software to obtain 61 DEGs. Among them, 28 genes were downregulated and 33 genes were upregulated. According to the gene logFC and *P* value difference genes, a volcano map was created (red is high expression, and green is low expression) as shown in Figure 1.

#### 3.1.2. GO and KEGG Pathway Enrichment Analyses

**Figure 2:**
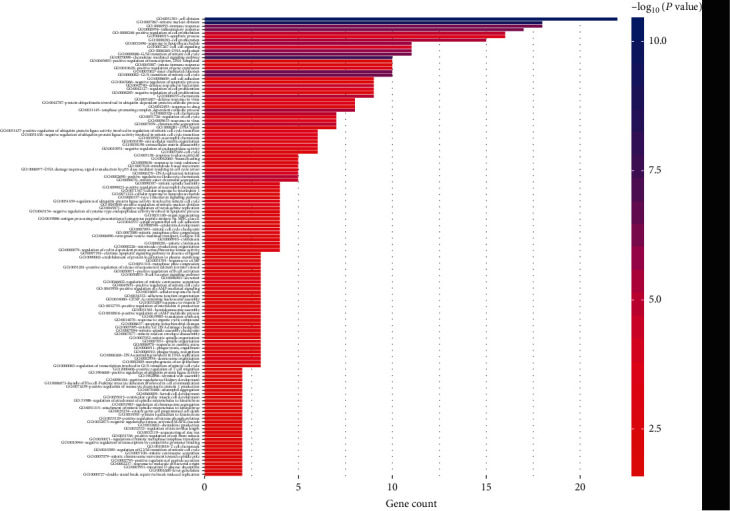
The main biological functions of DEGs.

**Figure 3:**
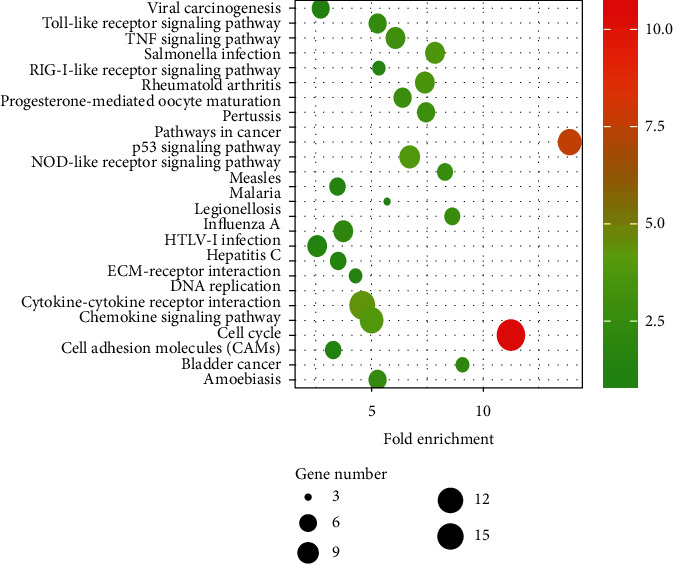
Significant pathway enrichment of DEGs.

The analysis indicated that the biological function of differential genes was mainly focused on the regulation of cell division, mitotic nuclear division, immune response, inflammatory response, positive regulation of cell proliferation, apoptosis, cell proliferation, response to lipopolysaccharide, DNA replication, G2/M transition of mitotic cell cycle, chemokine-mediated signaling pathway, DNA-templated, positive regulation of transcription, innate immune response, positive regulation of gene expression, etc. (Figure 2). KEGG pathway analysis showed that DEGs were primary involved in cell cycle, cytokine-cytokine receptor interaction, p53 signaling pathway, oocyte meiosis, chemokine signaling pathway, *Salmonella* infection, and other pathways (Figure 3).

#### 3.1.3. TCGA Database Query Gene Differential Expression

**Figure 4:**
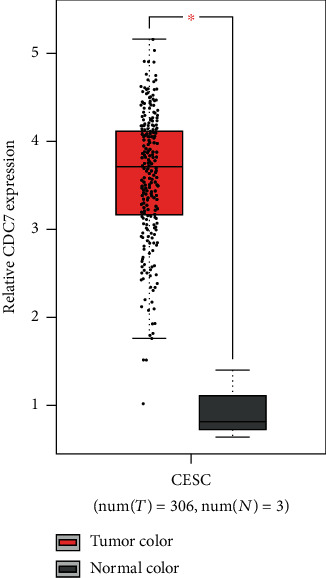
TCGA database showed that *CDC7* was highly expressed in cervical cancer tissues.

By using TCGA database to query the differential expression of differential genes in cervical cancer, it was found that *CDC7* was highly expressed in cervical cancer (Figure 4).

**Figure 5:**
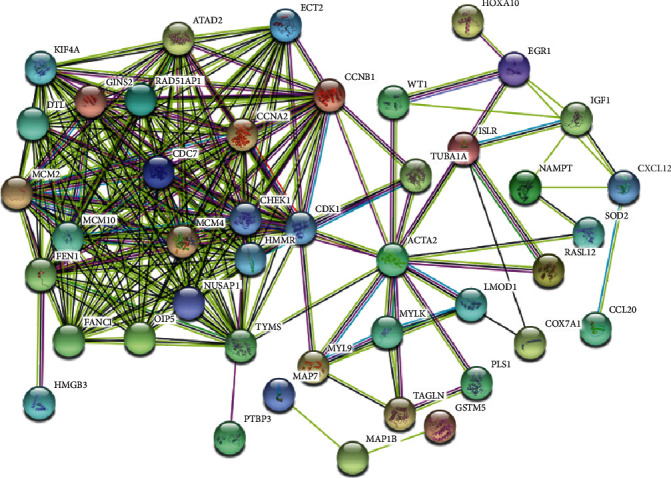
PPI network. Circles on behalf of genes and lines on behalf of the interaction of proteins between genes. Line color on behalf of evidence of the reciprocity between the proteins.

*CDC7*'*s PPI network*: protein interactions were analyzed online to ulteriorly investigate the interaction of *CDC7* with other genes (Figure 5).

### 3.2. Verification That *CDC7* Was Highly Expressed in Cervical Cancer Tissue

#### 3.2.1. Immunohistochemistry

**Figure 6:**
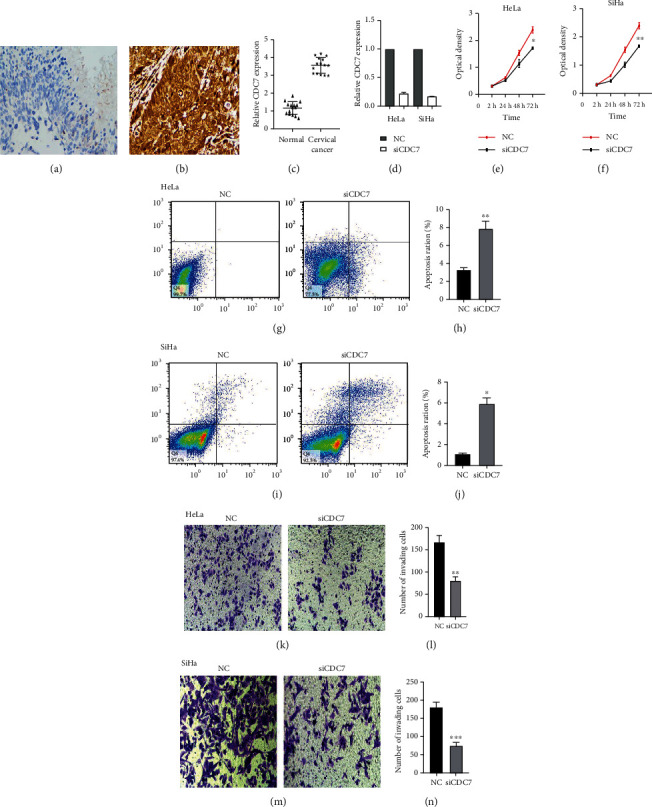
Characteristics and expression of *CDC7* in cervical cancer. Immunohistochemistry showed that *CDC7* was differentially expressed in (a) normal cervical epithelial tissues and (b) cervical cancer tissues. qRT-PCR detection shows the expression of *CDC7* in cervical cancer tissues and in the human normal cervical epithelial tissues. (c) The data are denoted by mean ± SD. ^∗^*P* < 0.05 vs. human normal cervical epithelial tissues. qRT-PCR detection shows the expression of *CDC7* in siCDC7 and siNC transfected cervical cancer cell lines (Hela and SiHa). (d) The data are denoted by mean ± SD. ^∗^*P* < 0.05 vs. NC. Downregulation *CDC7* suppressed cell proliferation. (e, f) Cell counting kit-8 (CCK-8) detection shows the cell proliferation of both (e) HeLa and (f) SiHa cells. The data are denoted by mean ± SD. ^∗^*P* < 0.05 and ^∗∗^*P* < 0.01 vs. NC. Downregulation *CDC7* suppressed cell invasion and promotes cell apoptosis. (g–j) Flow cytometry detection shows the cell apoptosis of both (g, h) HeLa and (i, j) SiHa cells. The data are denoted by mean ± SD. ^∗^*P* < 0.05 and ^∗∗^*P* < 0.01 vs. NC. (k–n) Shows the cell invasion of both (k, l) HeLa and (m, n) SiHa cells. The data are denoted by mean ± SD. ^∗∗^*P* < 0.01 and ^∗∗∗^*P* < 0.001 vs. NC.

*CDC7* protein was mainly localized to the nucleus in cervical tissue. Brown-yellow staining of the cells indicates positive for *CDC7* expression. Expression levels were calculated as the number of positive cells, that is, the percentage of positive cells in the 10 fields of view under the 200x microscope counted as a percentage under the microscope. In the end, <10% staining was judged as negative, and ≥10% was judged as positive. All tissue specimens were independently assessed by two pathologists (Figures 6(a) and 6(b)).

#### 3.2.2. qRT-PCR

Real-time quantitative RT-PCR results showed that the expression of *CDC7* in cervical cancer tissues was upregulated compared with normal cervical tissues, and the difference between the two groups was statistically significant (*P* < 0.05) (Figure 6(c)). Real-time quantitative RT-PCR results showed that the expression of *CDC7* in siCDC7 and siNC transfected cervical cancer cell lines (Hela and SiHa), and the difference between the two groups was statistically significant (*P* < 0.05) (Figure 6(d)).

### 3.3. Cell Function Verified the Effect of *CDC7* on Cervical Cancer Cell Line

#### 3.3.1. Proliferation

After transfection of si*CDC7*, qRt-PCR detection of *CDC7* expression level was significantly lower than the NC group. CCK-8 experiments showed that *CDC7* downregulation significantly inhibited cervical cancer cell line (Hela and SiHa) proliferation. These differences were statistically significant (*P* < 0.05; Figures 6(e)–6(f)).

#### 3.3.2. Apoptosis

Results of flow cytometry detection with Annexin V-FITC/PI double staining demonstrated that the apoptosis rates of si*CDC7* cells and control cells were 7.834 ± 0.619% and 3.190 ± 0.117% (Hela) and 5.393 ± 0.401% and 1.110 ± 0.140% (SiHa) after 36 h of transfection, respectively. These results showed that the apoptosis of cervical cancer cell lines (Hela and SiHa) was significantly induced by downregulation of *CDC7* (*P* < 0.05; Figures 6(g)–6(j)).

#### 3.3.3. Invasion

A Transwell assay was used to measure the number of cells stained by crystal violet. The results demonstrated cells invading matrigel and biofilm, which represented the invasive ability of the cells. The results showed that the number of cells invaded by si*CDC7* group was significantly lower than that of the control cell group (*P* < 0.05; Figures 6(k)–6(n)).

## 4. Discussion

With the implementation of the Human Genome Project, the use of sequencing and gene chips has rapidly developed. This growth has been further enhanced by the accessibility of TCGA, GEO, Oncomine, and other open databases that store gene expression data from a large number of normal and diseased tissues [10]. The integration of data from these databases allows researchers to further investigate new tumor biomarkers of significant research value [11, 12]. Based on the high morbidity and mortality of cervical cancer in gynecologic tumors, we used a comprehensive analysis method to evaluate new cervical cancer biomarkers from GEO datasets. Genetic screening revealed differential expression of *CDC7*, which may be related to the development of cervical cancer.

*CDC7* is overexpressed in many human tumor cell lines and tissues, such as ovarian cancer [13], colorectal cancer [14], lung cancer [15], salivary gland malignant tumor [16], and breast cancer [17]. However, expression in normal tissues and cell lines is very low or undetectable [13]. A correlation has been established between decreased expression of p53 and the overexpression of *CDC7* and DBF4 in primary breast cancer cells [6].

*CDC7* plays a crucial role in initiating DNA replication and DNA damage stress [18, 19]. There is also evidence that *CDC7* silencing increases tumor cell apoptosis independent of p53 expression [20, 21]. Additionally, overexpression of *CDC7* can also promote tumor cell tolerance and survival by multiple pathways [22]. Therefore, *CDC7* plays a fundamental role in cell proliferation, tumorigenesis, and malignant progression. Inhibition of such overexpression can then be used to treat certain cancers [6, 7]; therefore, *CDC7* has become a meaningful target for cancer treatment [23, 24].

Although some research on *CDC7* exists, its clinical significance and diagnostic value in cervical cancer have not been previously studied. Therefore, we first analyzed the expression of *CDC7* in cervical cancer tissue by immunohistochemistry and qRt-PCR and found that it was significantly higher than normal cervical tissue. Combined with *in vitro* cell experiments using the cervical cancer HeLa and SiHa cell lines, it was found that downregulation of *CDC7* expression in cervical cancer HeLa and SiHa cell lines significantly inhibited the proliferation and invasion of HeLa and SiHa cells and significantly increased the apoptosis of HeLa and SiHa cells. From this finding, it is clear that *CDC7* plays a significant role in the development about cervical cancer. In summary, our data suggest that *CDC7* may be an excellent tumor biomarker, and its clinical application warrants further study.

This study only found a single dataset and used in vitro experiments to verify the effectiveness of CDC7; the results are relatively single. In the future, it is necessary to further search and analyze more database information and use in vitro animal experiments to verify the effectiveness of CDC7. At the same time, we can further explore the possible mechanism of malignant tumors caused by high CDC7 expression.

## 5. Conclusion

This study suggests that, with comprehensive bioinformatics combined with clinical and cellular function analysis, *CDC7* is important to the development of cervical cancer. Targeting of this biomarker may improve the early diagnosis and treatment of cervical cancer.

## Data Availability

Readers can also make data available on request through the authors (wqw0116@126.com).
